# MiR-200b expression in breast cancer: a prognostic marker and act on cell proliferation and apoptosis by targeting Sp1

**DOI:** 10.1111/jcmm.12432

**Published:** 2015-01-30

**Authors:** YaSai Yao, Jian Hu, Zan Shen, RuYong Yao, ShiHai Liu, Yong Li, Hui Cong, XinGang Wang, WenSheng Qiu, Lu Yue

**Affiliations:** a Department of Oncology, Affiliated Hospital of Qingdao UniversityQingdao, China; b Molecular Cancer Biology and Translational Medicine Laboratory, Affiliated Hospital of Qingdao UniversityQingdao, China; c Department of Cancer Biology, University of Texas MD Anderson Cancer CenterHouston, TX, USA; d Department of Oncology, The Sixth People's Hospital, Shanghai Jiao Tong UniversityShanghai, China; e Central Laboratory, Affiliated Hospital of Qingdao UniversityQingdao, China; f Department of Galactophore Surgery, Affiliated Hospital of Qingdao UniversityQingdao, China

**Keywords:** MiR-200b, breast cancer, prognosis, Sp1, cell growth

## Abstract

MicroRNAs (miRNAs) have been identified as important post-transcriptional regulators involved in various biological and pathological processes of cells. In the present study, we investigated the roles and mechanisms of miR-200b in human breast cancer (BC). MiR-200b expression was carried out by qRT-PCR in human BC cell lines and clinical samples and the prognostic potential of miR-200b expression was further evaluated. *In vitro*, effects of miR-200b on BC cell proliferation, apoptosis and cell cycle distribution were tested by CCK-8 kit, flow cytometric analysis respectively. Luciferase assay and Western blot analysis were performed to validate the potential targets of miR-200b after the preliminary screening by employing open access software. We found that miR-200b was significantly down-regulated in both BC tissues and cell lines. The low expression of miR-200b was correlated with late TNM stage, negative oestrogen receptor and positive HER-2 status. Multivariate analysis showed that miR-200b expression was an independent prognostic predictor for BC patients. Integrated analysis identified Sp1 as a direct and functional target of miR-200b. Knockdown of Sp1 inhibited cell proliferation, induce apoptosis and act on cell cycle resembling that of miR-200b high expression. Our data demonstrates that miR-200b has potential to serve as prognostic biomarker and tumour suppressor for BC patients. As a direct and functional target of miR-200b, Sp1 and miR-200b both could be an exciting target for BC treatment strategy.

## Introduction

Worldwide, breast cancer (BC) is the second most prevalent malignancy and the fifth most common cause of cancer death 1. So far, the mechanism underlying the development of BC remains largely unclear, and the genetic and molecular alterations in this malignant disease are not fully understood. Thus, good understanding of the molecular mechanisms underlying BC development and progression is urgently needed.

In the past few years, small regulatory RNAs had gained enormous interests in cancer research. MicroRNAs (miRNAs), short (20–24 nt) non-coding RNAs, are involved in post-transcriptional regulation of gene expression in multicellular organisms by affecting both the stability and translation of mRNAs 2,3. Emerging evidence indicated that miRNAs are aberrantly expressed in different types of tumours 4,5 and participate in human tumourigenesis and/or metastasis by directly targeting oncogenes or tumour suppressor genes 6,7. Altered miRNA expressions, have been identified as modulators of tumour proliferation, apoptosis, and therapy resistance in BC 8. Indeed, specific miRNA dysregulation has been shown to correlate with BC 9. For instance, miR-21 is high expressed in BC tissue 10 while miR-132 is down-regulated in ductal carcinoma *in situ* of breast and acts as a tumour suppressor by inhibiting cell proliferation 11. Therefore, more extensive investigations are needed on the role of miRNAs, which are dysregulated in BC to develop novel avenues for targeted therapy.

Recently, the expression pattern and function of the miR-200 family has been widely studied in various cancers but remains controversial. The miR-200 family consists of five members: miR-200a, miR-200b, miR-200c, miR-429 and miR-141, which regulate the transcription factors Zeb1 and Ets-1 as well as Suz12, a subunit of the polycomb repressor complexes 12,13. It is a miRNA family with tumour-suppressive functions in a wide range of cancers, including BC 14, colorectal cancer 15, pancreatic cancer 16 and endometrial carcinoma 17.There is growing evidence to suggest that members of miR-200 family are implicated in diverse biological and pathological processes 18. To date, ground-breaking studies have established that miR-200b has been found to be involved in epithelial to mesenchymal transition, formation and maintenance of cancer stem cells, invasion of prostate cancer cells and gastric carcinoma 19. However, to our knowledge, its roles and the potential mechanisms in BC remain unclear. Hence, our study was aimed to identify the role of miR-200b in BC. We found that the expression of miR-200b was suppressed in both BC tissues and cancer cell lines. Furthermore, the low expression of miR-200b was associated with late TNM stage, negative oestrogen receptor (ER), positive human epidermal growth factor receptor 2 (HER-2) status. In addition, high expression of miR-200b inhibited cell proliferation, induce apoptosis and act on cell cycle by targeting Sp1, which was identified as a direct and functional target of miR-200b.

## Materials and methods

### Patients and tumour tissues

A total of 278 human BC tissues (BC) and matched normal adjacent breast tissues (NB) were obtained during the surgery at the Affiliated Hospital of Qingdao University and the Sixth People's Hospital, Shanghai Jiao Tong University between January 2003 and January 2012. None of the patients received chemotherapy or radiotherapy before the surgery. All 278 patients provided written informed consent for the use of their tissues and the study protocol was approved by the Ethics Committee of the local hospitals. Patient clinical information such as age, stage, ER, progesterone receptor (PR) and HER-2 status was provided from the archives of the above hospitals. The characteristics of the patients are shown in Table1.

**Table 1:** Correlation of patients' characteristic with the expressions of miR-200b

Variable	No. of cases	miR-200b expression		*P*-value
		Low	High	
Age				
<50	145	75	70	0.6347
≥50	133	65	68	
TNM stage				
I	96	37	59	0.0136*
II	108	59	49	
III	74	44	30	
ER				
Positive	177	79	98	0.0115*
Negative	101	61	40	
PR				
Positive	115	49	66	0.0575
Negative	163	91	72	
HER-2				
Positive	121	94	27	<0.001*
Negative	157	46	111	

* Statistically significant (*P* < 0.05).

### Cell lines and cultures

Human BC MDA-MB-231, SK-BR-3, MCF-7, MDA-MB-468 cell lines and a normal breast epithelial cell line HBL-100 were obtained from American Type Culture Collection and cultured in DMEM (Thermo Scientific HyClone, Beijing, China) supplemented with 10% foetal bovine serum, 100 U/mL penicillin, and 100 mg/ml streptomycin (Invitrogen, Carlsbad, CA, USA). All cells were incubated in 5% CO_2_ humid atmosphere at 37°C.

### Transfection

The miRNAs and siRNA against Sp1 were designed and synthesized by RiboBio (Ribobio Co, Guangzhou, China). Oligonucleotide transfection was done using Lipofectamine 2000 reagents according to the manufacturer's protocol. The BC cells were seeded in 12-well plates and were grown up to 60% confluence before the transfection. The final concentration of miR-200b mimic and anti-miR-200b was 50 nm. The sequence of siRNA targeting Sp1 was: AATGAGAACAGCAACAACTCC. All the RNA oligonucleotide sequences used were as follows: hsa-miR-200b mimics: 5′-CAUCUUACUGGGCAGCAUUGGA-3′; miR-NC: 5′-UUCUCCGAACGUGUCACGUTT-3′, miR-200b inhibitor: 5′-UCAUCAUUACCAGGCAGUAUUA-3′. inh-NC: 5′-CAGUACUUUUGUGUAGUACAA-3′. The RNA and proteins were extracted at 48 hrs after the transfection.

### Quantitative real-time qRT-PCR

Total RNA was isolated from BC tissues or cells using Trizol reagent (Invitrogen). MiR-200b and U6 were polyadenylated using poly-A polymerase based First-Strand Synthesis kit Takara Bio, KUSATSU, JAPAN following the manufacturer's protocol. To quantify the Sp1 and GAPDH mRNA levels, 1 ug of total RNA was subjected to first-strand cDNA synthesis for 15 min. at 37°C and 5s at 85°C using a PrimeScript RT Reagent kit (TaKaRa). The U6 or GAPDH were used as an endogenous control. The PCR primers were designed as follows: miR-200b sense, 5′-GCGGCTAATACTGCCTGGTAA-3′, and reverse, 5′-GTGCAGGGTCCGAGGT-3′; GAPDH sense, 5′-GCACCGTCAAGGCTGAGAAC-3′, and reverse, 5′-TGGTGAAGACGCCAGTGGA-3′; U6 sense, 5′-CGCTTCGGCAGCACATATACTA-3′, and reverse, 5′-CGCTTCACGAATTTGCGTGTCA-3′; Sp1 sense, 5′-CCTTCAGGGATTTCCAACTG-3′ and reverse, 5′-GTCCAAAAGGCATCAGGGTA-3′. The qPCR was performed with SYBR Green PCR master mix (TaKaRa) on the ABI 7500HT System. The relative fold expressions were calculated with the 2^−ΔΔCT^ method. All the qRT-PCR reactions were run in triplicate.

### Western blot analysis

Breast cancer cells were collected after 48 hrs treatment with 50 nM miR-200b mimic or miR-200b inhibitor and corresponding controls. Protein extraction, SDS-PAGE gel electrophoresis and blotting were performed. Briefly, proteins were extracted with RIPA buffer supplemented with protease inhibitors and quantified by the BCA method (Beyotime, Jiangsu, China). Lysates (25 μg) were separated on SDS-PAGE and then electrotransferred to nitrocellulose membranes (Whatman, Maidstone, UK). Membranes were blocked for 2 hrs at room temperature with 5% non-fat dried milk solution and then immunoblotted overnight at 4°C with primary antibodies against Sp1 and GAPDH (Santa Cruz Biotechnology Inc., Santa Cruz, CA, USA). After washing, the membranes were probed with HRP-conjugated secondary antibodies. Signals were visualized with Enhanced Chemiluminescence Plus Kit (GE Healthcare, Piscataway, NJ, USA) Values are an average of at least 3 independent experiments.

### Cell proliferation assay

Cells were seeded into 96-well plates (2 × 10^3^ cells/well) directly or 48 hrs after transfection and allowed to attach overnight. The proliferation of the cells was assayed at the indicated time points using a CCK-8 kit (Dojindo Laboratories, Kumamoto, Japan) according to the manufacturer's instructions. The assays were run in triplicate.

### Flow cytometric analysis of apoptosis

Cells were harvested directly or 48 hrs after transfection *via* ethylene diamine tetraacetic acid-free trypsinization. Early apoptosis rate was detected *via* flow cytometric analysis using an annexin V-fluorescein isothiocyanate apoptosis detection kit (Oncogene Research Products, Boston, MA, USA) as described by the manufacture's protocol. Values are an average of at least 3 independent experiments.

### Flow cytometric analysis of cell cycles

Cells were harvested directly or 48 hrs after transfection and washed with ice-cold PBS, and fixed with 70% ethanol overnight at −20°C. Fixed cells were rehydrated in PBS for 10 min. and subjected to PI/RNase staining followed by flow cytometric analysis using a FACScan instrument (Becton Dickinson, Mountain View, CA, USA) and CellQuest software (Becton Dickinson, San Jose, CA, USA) as described previously 20.

### Dual-luciferase assay

Co-transfection experiments were performed in 96-well plates. A total of 1 × 10^4^ cells were seeded per well in 200 ml medium. A total of 100 ng wild-type (WT) or mutant (MUT) reporter constructs were cotransfected with lipofectamine 2000 transfection reagent into the cells with 50 nM miR-200b or miR-NC according to the manufacturer's instruction. After 48 hrs, luciferase activity was measured with the Dual-luciferase reporter assay system (Promega, Madison, WI, USA) Firefly luciferase activity was then normalized to the corresponding renilla luciferase activity. Values are an average of at least 3 independent experiments.

### Statistical analysis

Comparisons between groups were analysed by the *t*-test and chi-squared test. Overall survival curves were plotted according to the Kaplan–Meier method, with the log-rank test applied for comparison. Survival was measured from the day of the surgery. Variables with a value of *P* < 0.05 by univariate analysis were used in subsequent multivariate analysis based on the Cox proportional hazards model. All differences were statistically significant at the level of *P* < 0.05. Statistical analyses were performed with the SAS 9.2 software.

## Results

### Expression of miR-200b is decreased in BC tissues

**Fig 1:**
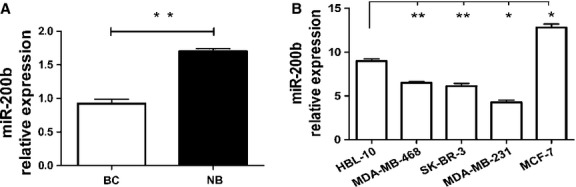
Relative expression levels of miR-200b in both breast cancer tissues and cell lines. (A) The relative expression levels of miR-200b in 278 pairs of breast cancer tissues and their matched corresponding non-tumourous tissues. BC, breast cancer tissues; NB, adjacent non-cancerous breast tissues. (B) The expression level of miR-200b in four breast cancer cell lines (MDA-MB-231, SK-BR-3, MDA-MB-468, MCF-7) and the normal breast epithelial cell line (HBL-10), respectively. *P < 0.05, **P < 0.01.

To gain insights into the role of miR-200b in breast tumourigenesis, we assessed its expression in 278 pairs of BC tissues and their matched non-tumourous breast tissues using qRT-PCR. As shown in Figure1A, the results showed that miR-200b expression was significantly decreased in BC tissues compared to that of matched non-tumourous breast tissues with the median fold change, 1.63 (*P* < 0.01). Therefore, the expression level of miR-200b was significantly decreased in BC tissues.

### Clinicopathologic significance of miR-200b in BC patients

Furthermore, we studied the correlation between miR-200b expression and clinical pathological characteristics of BC. The low miR-200b expression group showed a reverse correlation with higher incidence of late TNM stage (*P* = 0.0136), negative ER (*P* = 0.0115) and positive HER-2 status (*P* < 0.001) respectively. However, no significant differences were observed with respect to age, PR status in BC (shown in Table1).

**Fig 2:**
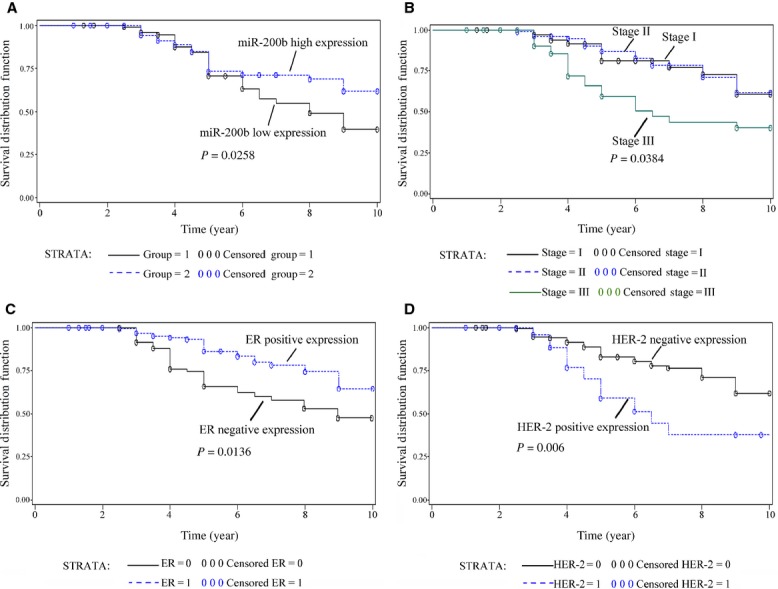
Kaplan–Meier survival curves for breast cancer patients by stratified univariate analysis. (A) Strata based on miR-200b expression (low and high expression). (B) Strata based on stage (stage I, II, III). (C) Strata based on ER status (positive and negative). (D) Strata based on HER-2 status (positive and negative).

To analyse the significance of miR-200b further in terms of clinical prognosis, a Kaplan–Meier survival analysis was performed (Fig.2). The results demonstrated that patients with low miR-200b expression had a significantly poorer prognosis than those with a high miR-200b expression. Our results indicated that expression levels of miR-200b were significantly associated with patients' overall survival.

Meanwhile, the final Cox's multivariate model revealed that reduced miR-200b level in tumours were independent predictors of shorter survival. TNM stage, ER and HER-2 status, as well as miR-200b, were independent prognostic factors as well (as shown in Table2). Taken together, these results showed that the miR-200b deregulation may play important roles in BC carcinogenesis, progression and was correlated with a worse prognosis.

**Table 2:** Univariate and multivariate analysis of factors associated with overall survival in breast cancer patients

Characteristics	Univariate analysis	Multivariate analysis	
	*P*-value	HR (95% CI)	*P*-value
Age	0.077	0.199 (0.036–1.104)	0.064
TNM stage	0.003*	2.303 (1.045–5.072)	0.038*
ER	<0.001*	0.180 (0.046–0.703)	0.014*
PR	0.342	1.387 (0.338–5.685)	0.649
HER-2	<0.001*	2.941 (1.589–5.445)	0.001*
miR-200b	0.027*	2.163 (1.098–4.264)	0.026*

* Statistically significant (*P* < 0.05).

HR, hazard ratio; CI, confidence interval.

### MiR-200b expression affects cell proliferation, apoptosis and cell cycle distribution *in vitro*

**Fig 3:**
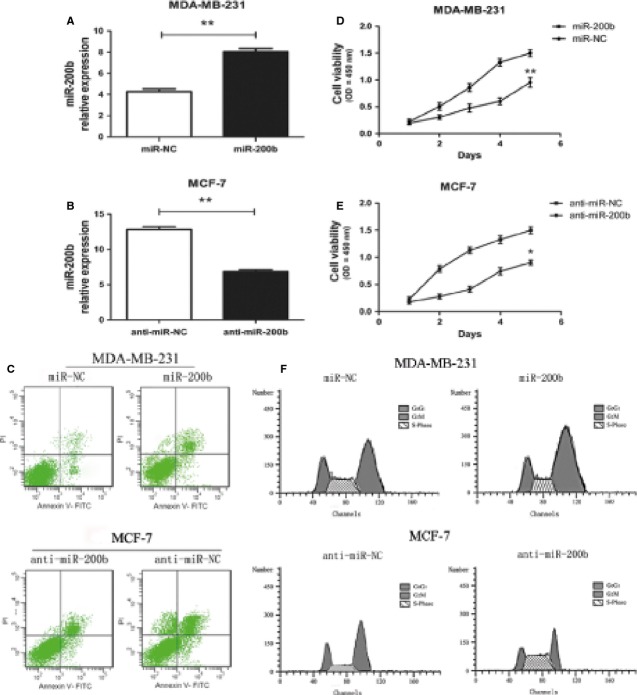
MiR-200b expression affects cell proliferation, apoptosis, and cell cycle distribution *in vitro*. (A and B) The expression level of miR-200b was tested in breast cancer cells after 48 hrs transfection with miR-200b mimics (miR-200b), anti-miR-200b (miR-200b inhibitor) and their respective NCs (50 nM) by qRT-PCR. (C) Cell apoptosis assays. (D and E) Cellular viability assay. (F) Cell cycle analysis. *P < 0.05, **P < 0.01.

In addition, the expression of miR-200b in four BC cell lines and normal breast epithelial cell line was determined by qRT-PCR. As a result, miR-200b was reduced in three BC cell lines (MDA-MB-231, SK-BR-3, MDA-MB-468) compared to the normal breast epithelial cell line HBL-100. And in the cell line MCF-7, however, the relative expression level for miR-200b was significantly increased (Fig.1B). To better understand the role of miR-200b in the development of BC, we transfected the cell line MCF-7 (as model cell lines for miR-200b high expression) with 50 nM miR-200b inhibitor, and MDA-MB-231 cells (as model cell lines for miR-200b low expression) were transfected with miR-200b mimics as well as their NCs, respectively. As shown in Figure3A, the miR-200b mimics caused a 3.62 folds increase in the miR-200b expression in MDA-MB-231 cell compared to their NCs (*P* < 0.01). Meanwhile, the miR-200b inhibitor decreased the miR-200b expression by 1.51-fold in MCF-7 (Fig.3B, *P* < 0.01). Furthermore, high expression of miR-200b significantly suppressed cell proliferation of MDA-MB-231 cell by 11.69 ± 2.17% compared with its control (*P* < 0.01, Fig.3D). And the miR-200b inhibitor promoted the growth of MCF-7 cells by 21.93 ± 2.07% respectively (*P* < 0.05, Fig.3E).

To analyse the mechanisms by which ectopic miR-200b expression inhibited cell proliferation, flow cytometric analysis of cell cycle and apoptosis was applied. As shown in Figure3C and F, enforced expression of miR-200b resulted in a dramatic increase of apoptosis in MDA-MB-231 cell from 4.07% ± 0.95% to 16.29% ± 1.64% compared with NC, but also an increased percentage of cells in G2/M phase from 11.69% ± 1.33% to 24.95% ± 2.31% and a decreased population in S phase from 39.67% ± 3.50% to 27.83% ± 1.10%. Opposite results were obtained in MCF-7 cells, the miR-200b inhibitor significantly reduced the apoptosis rate (25.37 ± 1.83% *versus* 6.17 ± 1.06%) as compared to that of the anti-miR-NC. Moreover, the inhibitor significantly reduced the proportion of G2/M phase from 22.54% ± 2.18% to 12.60% ± 1.45%, and increased the population of S phase from 23.75% ± 1.70% to 36.63% ± 1.49%.

### miR-200b down-regulate Sp1 by directly targeting its 3′UTR

**Fig 4:**
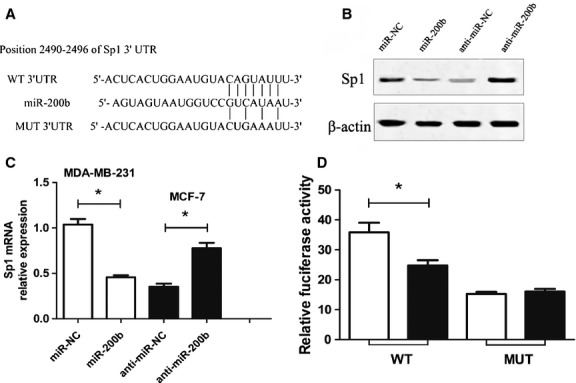
miR-200b down-regulate Sp1 by directly targeting its 3′UTR. (A) A putative binding sequences of miR-200b in the Sp1′UTR. Mutation was generated in the Sp1 3′UTR by mutating 3 nt that is recognized by miR-200b. Either wild-type (WT) or mutant (Mut) Sp1 3′UTR was subcloned into the dual-luciferase reporter vector. (B) The effects of miR-200b or anti-miR-200b on the expression of Sp1 by western blot analysis. (C) The effects of miR-200b or anti-miR-200b on the expression of Sp1 by qRT-PCR method. (D) Luciferase assay in MDA-MB-231 cells cotransfected with miR-200b and a luciferase reporter containing the Sp1 3′ UTR (WT) or a mutant (Mut). *P < 0.05.

To explore the molecular mechanism of miR-200b in BC, we searched for putative protein-coding gene targets of miR-200b. By employing open access softwares (TargetScan, miRBase Targets and PicTarget), transcription factor Sp1 was chosen as a preferred candidate target gene of miR-200b because of the 3 complementary sites of miR-200b in its 3′-UTR (Fig.4A).

Furthermore, the MDA-MB-231 cell was transfected with miR-200b and then examined for Sp1 expression by qRT-PCR and western blot. As shown in Figure4C, the miR-200b transfection led to an obvious decrease in SP1 mRNA expression (*P* < 0.05). On the contrary, transfection of anti-miR-200b resulted in an up-regulation in the Sp1 mRNA expression in MCF-7 (*P* < 0.05). Similarly, western blot analysis showed that enforced expression of miR-200b triggered a silencing effect on the endogenous Sp1 protein expression (Fig.4B). Those results suggested that the Sp1 expression is regulated by miR-200b in BC.

To further confirm the possibility that miR-200b targets Sp1, the target sequences of Sp1 3′UTR (WT) or the mutant sequence (mutant type, MUT) were cloned into the luciferase reporter vector, respectively. Luciferase reporter assays showed that the miR-200b significantly decreased the firefly luciferase activity in the reporter with WT 3′UTR, but had no effect on the mutant of Sp1-3′UTR (*P* < 0.05, Fig.4D). Taken together, these results suggested that miR-200b down-regulated Sp1 expression by directly targeting its 3′UTR.

### Sp1 was up-regulated in BC tissues and was inversely correlated with miR-200b levels

**Fig 5:**
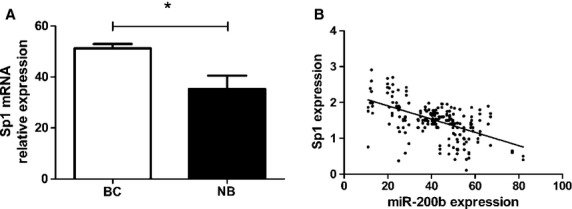
Relative expression levels of miR-200b and Sp1 up-regulation in breast cancers. (A) Sp1 was detected using qRT-PCR in 278 pairs of breast cancer (BC) tissues and their matched non-cancerous breast tissues (NB). (B) The reverse relationship between Sp1 and miR-200b expression was explored by Spearman's correlation in BC tissues. *P < 0.05, **P < 0.01.

As miR-200b is down-regulated in BC and targets Sp1 by binding to its 3′UTR, we next determined whether Sp1 mRNA expression is negatively associated with miR-200b levels in the BC tissue samples. We found that the average expression level of Sp1 was significantly higher in BC tissues than in matched normal tissues (*P* < 0.05) (Fig.5A). In addition, a statistically significant inverse correlation was observed by Spearman's correlation analysis between expression levels of miR-200b and Sp1 mRNA in BC tissue (*r* = −0.6531, *P* < 0.0001, Fig.5B).

### Sp1 is involved in miR-200b–induced suppression of BC cell growth

**Fig 6:**
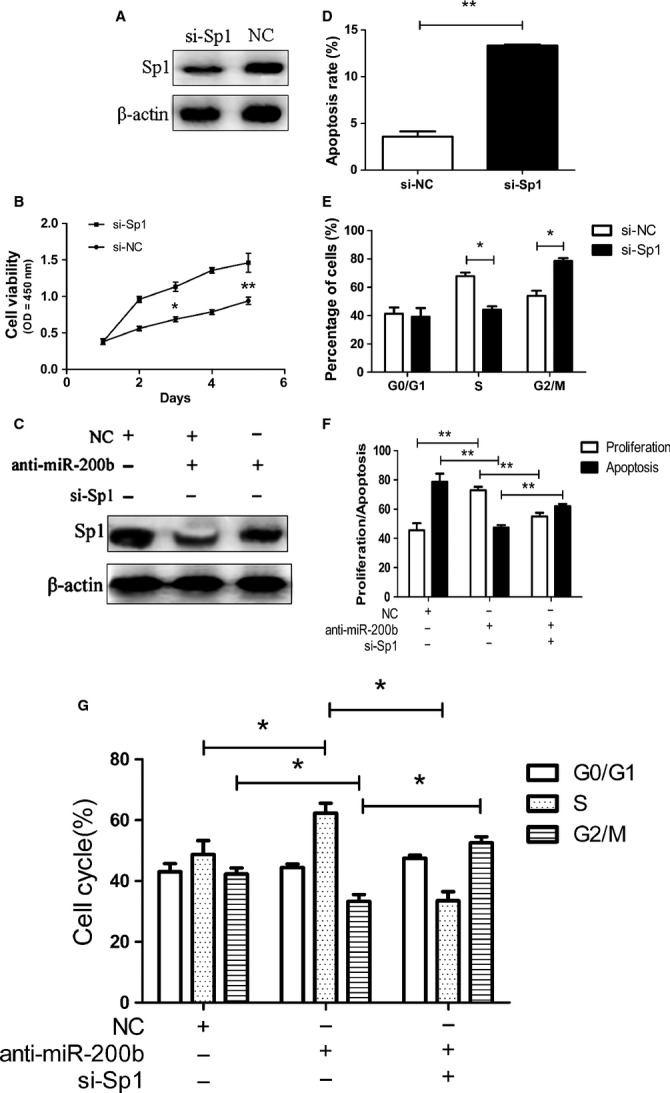
Involvement of Sp1 in miR-200b-induced suppression of breast cancer cell growth. (A) Silencing of Sp1 was confirmed by Western blot in MDA-MB-231 cell after transfection with specific si-Sp1. β-actin served as an internal control. (B, D and E) apoptosis, proliferation and cell cycle assays were carried out in MCF-7 cells after transfection with negative control (NC) or si-Sp1. (C) Western blot analysis was used to detect the Sp1 expression in MCF-7 cells after transfection with miR-200b inhibitor, si-Sp1, or NC. β-actin served as an internal control. (F) MCF-7 cells after transfection with miR-200b inhibitor, si-Sp1, or NC were subjected to proliferation, apoptosis assays. (G) MCF-7 cells after transfection with miR-200b inhibitor, si-Sp1, or NC were subjected to cell cycle assays. *P < 0.05, **P < 0.01.

To further examine whether miR-200b exerts its tumour suppressor function through dysregulation of Sp1, we performed functional analyses on Sp1. First, specific siRNAs against Sp1 were exploited to knockdown Sp1 expression in MDA-MB-231. Western blotting analysis confirmed that si-Sp1 significantly reduced the expression of Sp1 protein (Fig.6A). Cell proliferation and flow cytometric analysis showed that si-Sp1 could inhibit BC cell growth, increase cancer cell apoptosis, affect cell cycle (Fig.6B, D and E), which resembled the inhibitory effects of miR-200b on the BC cell. To determine whether deregulation of Sp1 is involved in regulation of cell growth, apoptosis and cell cycle by miR-200b, we cotransfected MCF-7 cells with miR-200b inhibitor and si-Sp1 as described in Figure6C, the expression of Sp1 was confirmed by Western blotting. Interestingly, we found that the proliferation, apoptosis and cell cycle promoting effects of anti-miR-200b were partially attenuated by si-Sp1 (Fig.6F and G). Taken together, these data demonstrated that Sp1 was a functional target of miR-200b and involved in miR-200b-induced suppression of BC cell growth.

## Discussion

In this study, we found that miR-200b was frequently down-regulated in both BC tissues and cell lines. And the deregulated miR-200b was correlated with late TNM stage, negative ER and positive HER-2 status. In addition, high expression of miR-200b could affect cell proliferation, apoptosis and cell cycle distribution of BC. Furthermore, Sp1 was characterized as the functional target of miR-200b. Accordingly, these results suggest that miR-200b might be useful as a prognostic marker to predict survival and a novel tumour suppressor miRNA in BC.

MiRNAs have been reported to play important roles in carcinogenesis and tumour progression 21, which function as classical oncogenes or tumour suppressor genes. Accumulating evidence suggests that there are correlations between miRNA expression and clinical recurrence, development of metastases and/or survival 22,23. MiR-200b, a member of miRNA-200 family, functions as tumour suppressor in a wide range of human malignances, including BC, colorectal cancer, pancreatic cancer 14–16. In our study, we found that miR-200b was down-regulated in both BC tissues and cell lines compared with their corresponding non-tumourous controls. And the deregulated miR-200b in BC was correlated with late TNM stage, negative ER and positive HER-2 status. In a well-characterized series of ovarian carcinomas, low tumoural expression of miR-200 family was significantly associated with high b-tubulin III protein content and had a trend towards poor progress-free survival (PFS) 24, suggesting the potential significance of miR-200 family members as new biomarkers. Recent study 25 also reported that miR-200b was a valuable marker for gastric cancer prognosis and played an important role in the development and progression of human gastric cancer. Together with our results, these data suggest that miR-200b may have potential to serve as prognostic biomarker for various cancers including BC. Our results indicate that the expression level of miR-200b might provide useful information in the evaluation prognosis and follow-up schedule guiding for BC patients. Meanwhile, our discoveries have also provided a brand new thinking to find other new prognostic and predictive factors.

So far, there has been firmly established that miRNAs control various key cellular processes such as proliferation 26, apoptosis 27, differentiation 28, development and were implicated in cancers. Previous study reported that miR-200 high expression inhibited tumour growth and metastasis in lung adenocarcinoma 29. Moreover, miR-200b showed tumour-suppressive activity in human malignant glioma 30. In our study, high expression of miR-200b significantly suppressed cell proliferation of MDA-MB-231 cell by 11.69 ± 2.17% compared with its control. Furthermore, enforced expression of miR-200b resulted in a dramatic increase of apoptosis in MDA-MB-231 cell compared with its negative control, but also an increased percentage of cells in G2/M phase and a decreased population in S phase, which was accordance with the above studies. Our data confirmed the regulatory function of miR-200b in cancer development and progression step further. However, the downstream signalling pathways or some specific factors which acts on these biological functions of tumour cells, or identifying and elucidating the possible molecular mechanisms underlying them, still need further investigation.

In view of the vital importance of miR-200b, we further explored the molecular mechanisms underlying BC cell biological behaviour by the regulation of miR-200b. Supporting this notion, we and colleagues used TargetScan algorithm to search for putative protein-coding gene targets of miR-200b, especially for those that have the abilities to promote tumour cell proliferation and apoptosis. Based on this rationale, four candidate genes GATA4, ROCK1, SP1 and WNT1 were selected in our prophase investigation and finally validated Sp1 was one of targets of miR-200b in BC (data not shown). Sp1, a member of Sp/KLF (Krüppel-like factor) transcription factors family, which was expressed highly in numerous cancers and important for a variety of physiological processes, including angiogenesis, cell cycle progression, inflammation, and senescence 30–32. Until now, apart from our study, very little information is available regarding the effect on Sp1 in BC. Only very recently, Natasha Kolesnikoff *et al*. reported that Sp1 binds to the miR-200b proximal promoter and activates miR-200 expression in epithelial cells, which has implications in cancer early differentiation and for designing interventions to prevent cancer metastasis 32. In agreement with the above study, our data showed that high Sp1 expression was associated with low miR-200b levels in BC (*P* < 0.001) and Sp1 is a direct functional target of miR-200b. Our evidence suggested that dysregulation of Sp1 signalling pathway by miRNAs is an important mechanism underlying cancer procession, and may serve as potential treatments for modulating this pathway in cancers. On the other side, as we all know, Sp1 is responsible for up-regulation of housekeeping genes (VEGF, uPA, uPAR and EGFR), which participate in tumour cell angiogenesis and metastasis 33. Since down-regulation of Sp-1 leads to inhibition of cell proliferation, transactivation gene expression might be a possible mechanism for preventing the tumour cell invasion and metastasis. Hence, our work provided a clue that, whether it may prevent tumour formation, proliferation, migration and invasion by inhibiting the expressions of Sp1′s housekeeping genes. And also, the specific molecular mechanism on Sp1 was still needed to be implicated. What's more, there has a long way for Sp1 used as a molecular-target in the therapy for BC.

In conclusion, miR-200b may not only be a potential prognostic marker for predicting the survival and relapse but may be also a tumour suppressor modulator in BC. As shown in this research, enforced miR-200b expression significantly inhibited BC cell growth, induce apoptosis and arrest cell cycle by targeting Sp1. Better understanding of the precise molecular mechanism for aberrant miR-200b signalling pathway may help design effective therapeutic modality to control BC. Therapeutic strategies that silence Sp1 may have the possibility to benefit BC patients.
